# Cryptochrome-mediated integration of circadian rhythms into seasonal cycles in the silkworm *Bombyx mori*

**DOI:** 10.3389/fphys.2026.1953101

**Published:** 2026-09-08

**Authors:** Daniel J. Brady, Federica Sandrelli

**Affiliations:** 1 Fraunhofer Institute for Molecular Biology and Applied Ecology—IME, Branch for Bioresources, Gießen, Germany; 2 Department of Biology, University of Padova, Padova, Italy

**Keywords:** *Bombyx mori*, circadian clock, cryptochrome, diapause, eclosion, hatching, photoperiodism, seasonal timing

## Abstract

The domesticated silkworm *Bombyx mori* is both a major sericultural species and a powerful model for studying how environmental cues are converted into developmental decisions. A central seasonal trait in this species is embryonic diapause, a maternally programmed developmental arrest whose incidence is shaped by genetic background and environmental conditions experienced during the maternal generation. Recent genome-editing studies have provided new functional evidence for the role of circadian clock genes in this process, particularly the two silkworm cryptochromes, BmCRY1 and BmCRY2. BmCRY1 belongs to the *Drosophila*-type CRY lineage and retains features consistent with a light-input function, whereas BmCRY2 belongs to the mammalian-type insect CRY lineage and displays structural motifs expected for a nuclear transcriptional repressor. Loss of *Bmcry1* disrupts embryonic hatching and adult eclosion rhythms and impairs photoperiodic modulation of diapause, while temperature-dependent diapause induction under constant darkness is largely retained. By contrast, *Bmcry2* mutants show reduced temperature-dependent diapause induction, although the direct role of BmCRY2 in the silkworm clockwork remains unresolved. Together, available evidence suggests that BmCRY1 and BmCRY2 act through distinct clock-related entry points that ultimately influence, directly or indirectly, the neuroendocrine pathway controlling diapause hormone release and maternal diapause commitment. These findings place silkworm cryptochromes at a key interface between environmental sensing, circadian timing and seasonal developmental control, with potential implications for sericulture.

## 1 Introduction

*Bombyx mori* is a domesticated lepidopteran derived from the wild mulberry silkmoth *Bombyx mandarina*. Selected for silk production for thousands of years, it remains a major sericultural species and a classical model for lepidopteran genetics and physiology (Garel, 1982; Goldsmith et al., 2005; Xia et al., 2014). Its strain diversity, genetic resources and genome-editing tools facilitate studies linking development, circadian timing and seasonal physiology with traits of practical relevance, including silk production and pupal weight (Ma et al., 2019).

Circadian clocks are endogenous timing mechanisms that generate approximately 24-h rhythms in molecular, cellular, physiological, and behavioral traits, even in the absence of external cues (free-running conditions). They are synchronized by environmental signals, particularly light-dark (LD) cycles, allowing organisms to anticipate daily environmental changes. In *B. mori*, the circadian system has been implicated in rhythmic developmental and behavioral outputs, including egg hatching and adult eclosion (Saunders, 2002; Brady et al., 2021).

At the molecular level, circadian clocks are generally based on interlocked transcription-translation feedback loops (TTFLs). In the main TTFL, CLOCK and CYCLE/BMAL1 activate the expression of negative regulators like PERIOD (PER) and TIMELESS (TIM), which later repress CLOCK: CYCLE/BMAL1-mediated transcription and generate daily molecular oscillations (Patke et al., 2020). In Lepidoptera, this general organization is retained, but differs from the canonical *Drosophila melanogaster* model in the presence of two CRYPTOCHROME proteins (CRYs) with distinct functions: a *Drosophila*-like CRY1, primarily associated with light input and TIM degradation, and a mammalian-like CRY2, which acts together with PER and TIM as a transcriptional repressor within the main TTFL (Zhu et al., 2005, 2008; Yuan et al., 2007; Zhang et al., 2017; Brady et al., 2021).

Early RNAi studies provided initial evidence for clock-gene function in the *B. mori* circadian system (Sandrelli et al., 2007a). Since 2019, TALEN- and CRISPR/Cas9-mediated genome editing has generated stable loss-of-function mutant lines for *Bmper*, *Bmtim*, *BmClk*, *Bmcyc*, *Bmcry1* and *Bmcry2* (Ikeda et al., 2019, 2021; Nartey et al., 2021; Homma et al., 2022; Tobita and Kiuchi, 2022, 2024; Yuan et al., 2023; Brady et al., 2025). Disruption of the core clock genes *Bmper*, *Bmtim*, *BmClk* or *Bmcyc* caused arrhythmic embryonic hatching and/or adult eclosion under constant conditions, such as constant darkness (DD), confirming their requirement for normal circadian rhythmicity in *B. mori* (Ikeda et al., 2019, 2021; Nartey et al., 2021; Brady et al., 2025; Tobita and Kiuchi, 2026).

A key seasonal trait in *B. mori* is embryonic diapause, a programmed developmental arrest that allows insects to survive unfavorable seasons (Denlinger, 2002). Its control is also important for egg storage, synchronized hatching, and planning of rearing cycles. Diapause has a complex relationship with voltinism, i.e., the number of generations completed within a year (one in monovoltine, two in bivoltine and several in polyvoltine strains). Voltinism is a multilocus trait, and its relationship with diapause is shaped by interactions between genetic background and environmental cues (Morohoshi, 1969; Grenier et al., 2004; Liu et al., 2025; Zheng et al., 2025). Environmental modulation of diapause is particularly evident in bivoltine strains (Shimizu, 2024; Zheng et al., 2025).

Although in *B. mori* diapause occurs during embryogenesis, its induction is maternally programmed. In bivoltine strains, photoperiodic and thermal cues experienced by the mother during embryonic and larval development, together with nutritional conditions and humidity, influence whether the female will later produce diapause or non-diapause eggs (Kogure, 1933; Shimizu, 2024). High temperature and long days during embryogenesis and short days during larval development promote diapause-egg production in the subsequent generation. This transgenerational and genotype-dependent organization makes *B. mori* an interesting model for studying how environmental cues are perceived, integrated and converted into endocrine signals that determine developmental fate across generations.

Among the environmental cues controlling diapause, photoperiod has been extensively studied in *B. mori*. As in other photoperiodic insects, daily LD cycles provide a predictable seasonal cue, linking diapause induction with photoreception, time measurement, and endocrine regulation (Koštál, 2011; Dolezel, 2015; Shimizu, 2024). In *B. mori*, the identity of the photoreceptor(s) mediating diapause-relevant light signals has long been a central question. Physiological and spectral studies indicated that diapause-relevant light information is perceived in the brain, with maximal sensitivity in the blue-green range (about 411–547 nm) and little response to wavelengths above ~580 nm (Shimizu and Hasegawa, 1988; Shimizu, 2024). Nutritional evidence further implicated retinal-based photopigments, since vitamin A/carotenoid availability affects the photoperiodic response and retinal derivatives have been detected in the larval brain (Shimizu et al., 1981; Shimizu and Kato, 1984; Hasegawa and Shimizu, 1987, 1988; Shimizu and Hasegawa, 1988; Shimizu, 2024). These observations led to the identification of the cerebral opsin Boceropsin as a candidate photoperiodic receptor, although its functional role remains unresolved (Shimizu et al., 2001; Shimizu, 2024).

However, opsin-based photoreception may not be the only route for diapause-relevant light input in *B. mori*. Because photoperiodic responses require the interpretation of daily LD information, silkworm diapause has also been studied in relation to circadian clock mechanisms (Saunders, 2020; Shimizu, 2024). A functional link between the circadian clock and photoperiodic regulation of seasonal reproduction has been established in the monarch butterfly *Danaus plexippus*, where loss of *Clk*, *Bmal1* or *Cry2* abolished photoperiodic reproductive responsiveness (Iiams et al., 2019). Nevertheless, circadian involvement in photoperiodism appears to vary across Lepidoptera. Hourglass-like mechanisms, sometimes with circadian contributions, have been reported in *Pieris brassicae*, *P. melete* and *Pseudopidorus fasciata* (Veerman et al., 1988; Wei et al., 2001; Xiao et al., 2009). In *Ostrinia nubilalis* circadian involvement has been associated with diapause induction and hourglass-like timing with diapause termination (Skopik and Takeda, 1986).

This diversity highlights the need to clarify how circadian and photoreceptive mechanisms contribute to diapause regulation in *B. mori*. In this context, BmCRY1 is particularly relevant because it is a *Drosophila*-like CRY, related to the blue-light-sensitive circadian photoreceptor characterized in *Drosophila*, and may therefore link light input, circadian timing, and photoperiodic diapause regulation (Emery et al., 1998, 2000; Stanewsky et al., 1998; Saunders, 2012; Goto, 2022; Tobita and Kiuchi, 2024).

Here, we discuss current evidence for BmCRY1 and BmCRY2 in circadian and diapause-related phenotypes, and consider their implications for photoreception, endocrine output, developmental memory and strain-dependent diapause regulation in *B. mori*, a species of major sericultural importance.

## 2 Background

### 2.1 BmCRY1 and BmCRY2 in the silkworm circadian clock: evolutionary and structural divergence and temporal regulation

**Figure 1:**
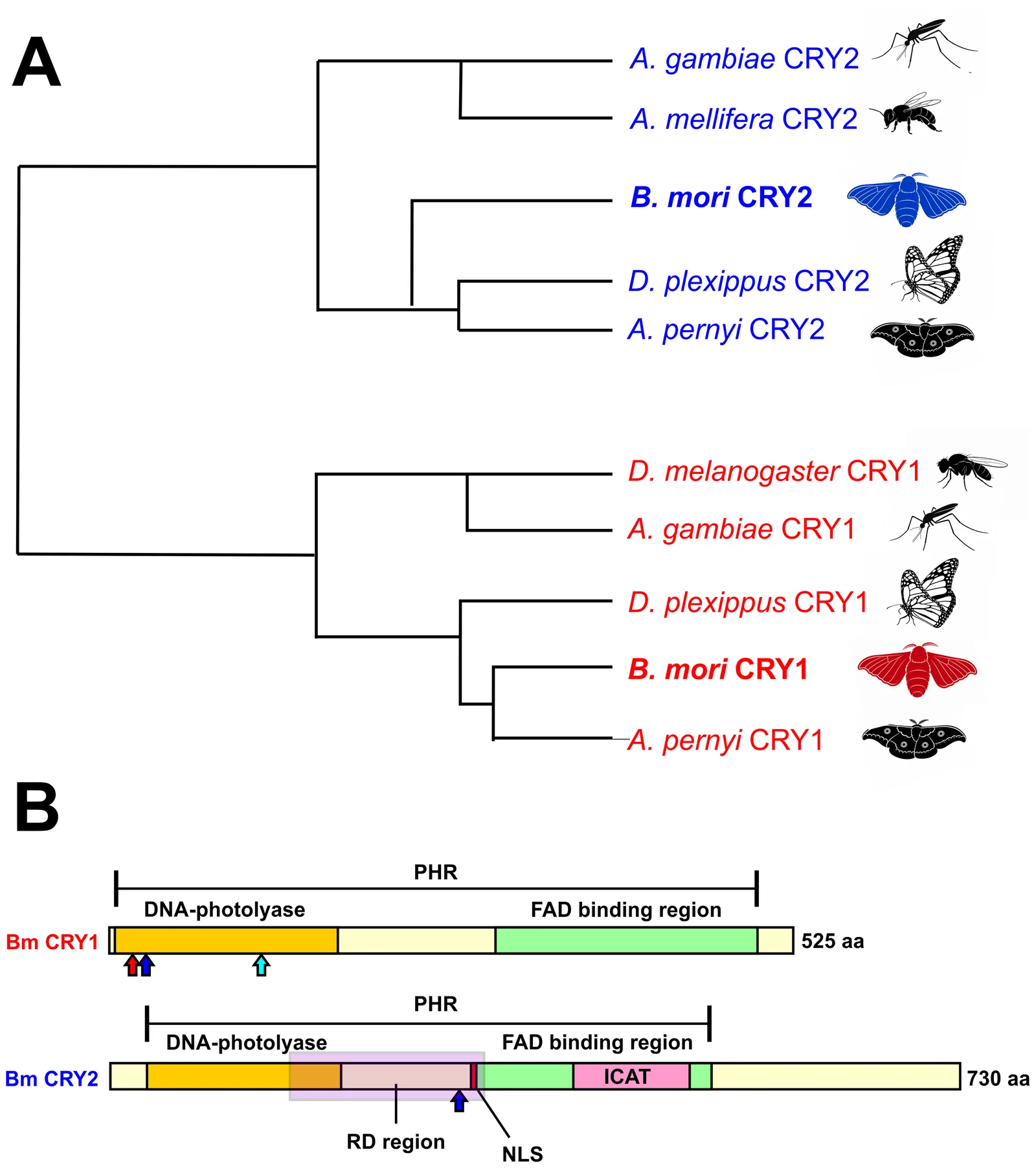
Evolutionary and structural organization of *B. mori* CRYs. **(A)** Simplified phylogenetic tree of representative insect CRYs, showing BmCRY1 and BmCRY2 within the CRY1 and CRY2 lineages, respectively. The topology is schematically redrawn from (Tobita and Kiuchi, 2024). **(B)** Schematic domain organization of BmCRY1 and BmCRY2, with the approximate positions of available genome-edited mutant alleles. Protein maps were drawn using BmCRY1 RefSeq NP_001182628.1 and BmCRY2 RefSeq NP_001182627.1, together with NCBI conserved-domain annotations and the BmCRY2 domain organization reported in (Homma et al., 2022; Tobita and Kiuchi, 2024). PHR indicates the conserved photolyase homology region; the orange box represents the DNA-photolyase domain, and the green box indicates the FAD-binding region. In BmCRY2, a semi-transparent purple box represents the RD region, comprising three repression-related domains. The predicted nuclear localization signal (NLS) and the inhibition of CLOCK-ARNTL-mediated transcription (ICAT) domain are indicated by red and magenta boxes, respectively. Colored arrows mark mutant allele positions: in BmCRY1, the red arrow indicates the *Cry1^−/−^* allele reported in (Qiu et al., 2023; Yuan et al., 2023), the blue arrow indicates *Δcry1_5317* described in (Homma et al., 2022), and the light-blue arrow indicates the *cry1Δ^8^* allele described in (Tobita and Kiuchi, 2024). In BmCRY2, the blue arrow indicates *Δcry2_5731* described in (Homma et al., 2022). Species names are abbreviated according to standard nomenclature. Insect silhouettes were manually traced from reference photographs and refined using AI-assisted smoothing. Domain boundaries, allele positions and phylogenetic relationships are shown schematically and are not drawn to scale.

Phylogenetic analysis places BmCRY1 and BmCRY2 in two distinct insect cryptochrome lineages. BmCRY1 clusters with *Drosophila*-type CRYs, while BmCRY2 clusters with mammalian-type insect CRYs (Tobita and Kiuchi, 2024) (**Figure 1A**). This separation is consistent with the presence of both CRY lineages across Lepidoptera, including Bombycoidea, and with the functional distinction between CRY1 and CRY2 characterized in *D. plexippus* (Zhu et al., 2005, 2008; Yuan et al., 2007; Brady et al., 2021). Both BmCRY1 and BmCRY2 retain a conserved photolyase homology region (PHR), extending over most of BmCRY1 and over the N-terminal half of BmCRY2 (**Figure 1B**). This PHR includes DNA-photolyase-related motifs and residues involved in binding flavin adenine dinucleotide (FAD), with the FAD-binding region embedded within the same photolyase-like core. Although CRY proteins derive from photolyases, animal CRYs generally no longer function as DNA-repair enzymes but retain this flavoprotein core for light-dependent and/or clock-related functions (Mei and Dvornyk, 2015; Michael et al., 2017). The best-characterized insect example is *D. melanogaster* CRY, in which light activation promotes TIM degradation and clock resetting (Emery et al., 1998, 2000; Stanewsky et al., 1998; Patke et al., 2020).

Despite this shared PHR, BmCRY1 and BmCRY2 differ in predicted regions associated with clock regulation. BmCRY2 contains conserved repression-related motifs, including repression domain (RD) regions and an ICAT region, named for inhibition of CLOCK–ARNTL/BMAL1-mediated transcription, as well as a predicted nuclear localization signal (NLS). These features are not conserved in BmCRY1 (Tobita and Kiuchi, 2024). Together, phylogeny and domain organization support a functional distinction in *B. mori*, with BmCRY1 resembling a CRY1-type light-input protein, and BmCRY2 displaying features expected for a CRY2-type nuclear repressor within the clockwork.

Daily expression of *Bmcry1* and *Bmcry2* has been examined in several studies. In larval heads under LD, *Bmcry1* mRNA peaked at ZT 1-5, similar to *D. melanogaster cry* mRNA expression (Emery et al., 1998; Tobita and Kiuchi, 2024). In embryos, both genes showed more variable profiles across studies, likely reflecting differences in strains, developmental stages, sample types and lighting regimes (Tao et al., 2017; Brady et al., 2021; Homma et al., 2022; Qiu et al., 2023), although *Bmcry2* generally showed higher expression between ZT 16 and ZT 24 under LD (Tao et al., 2017; Homma et al., 2022). At the protein level, both BmCRY1 and BmCRY2 oscillated in late embryos under LD, reaching maximal levels around ZT 24/0 (Tao et al., 2017).

In *D. melanogaster*, V/P-box elements contribute to rhythmic *cry* transcription through VRILLE and PDP1 (Glossop et al., 2003; Hao Zheng et al., 2008). Similar regulation was suggested for *Bmcry1*, based on its altered expression in core clock mutants (Tobita and Kiuchi, 2024). However, to our knowledge, V/P-box elements have not yet been characterized in the regulatory regions of *Bmcry1* or *Bmcry2*, and their contribution to the observed expression patterns remains unknown.

### 2.2 Genome-edited mutants reveal distinct timing-related roles for BmCRY1 and BmCRY2

The functional divergence suggested by phylogeny and domain organization is supported by genome-edited mutants. Independent *Bmcry1* loss-of-function alleles have provided convergent evidence for BmCRY1 involvement in circadian and photoperiodic phenotypes (**Figure 1**; **Table 1**). In a TALEN-mediated knockout line, embryos were exposed to 12:12 LD for six days followed by DD, or to LL or DD throughout. Wild-type embryos hatched rhythmically after LD entrainment and free-ran in DD with a period of ~28 h, whereas *Bmcry1* mutants were arrhythmic under all conditions (Yuan et al., 2023).

**Table 1:** Structural features and functional evidence for BmCRY1 and BmCRY2 in circadian and diapause-related phenotypes in *Bombyx mori*.

Feature/phenotype	BmCRY1	BmCRY2
Clock-related structural features	Conserved PHR/DNA-photolyase and FAD-binding regions (Homma et al., 2022; Tobita and Kiuchi, 2024).	Conserved PHR, including DNA-photolyase and FAD-binding regions. NLS, ICAT and repression-related domains consistent with a clock repressor-type CRY (Homma et al., 2022; Tobita and Kiuchi, 2024).
Circadian molecular features	Cycling *Bmcry1* expression has been reported in wild-type larval heads and embryos. A peak of expression was reported in larval heads at ZT 1-5; embryonic rhythmicity and phase vary among studies. BmCRY1 protein levels oscillate in late embryos under LD, reaching a peak around ZT 24/0 (Tao et al., 2017; Homma et al., 2022; Qiu et al., 2023; Tobita and Kiuchi, 2024). *Cry1^−/−^* affects clock-gene expression and hatching-associated neuropeptide/hormone signaling in embryos (Qiu et al., 2023; Yuan et al., 2023).	Cycling *Bmcry2* expression has been detected in wild-type embryos, with a peak between ZTs 16 and 24, under LD. *Bmcry2*rhythmicity under DD differs among studies. BmCRY2 protein levels oscillated in late embryos in LD, with maximal levels at ZT 24/0 (Tao et al., 2017; Homma et al., 2022; Qiu et al., 2023).
Circadian behavior	*Cry1^−/−^* alters embryonic hatching rhythmicity and photic entrainment (Homma et al., 2022; Tobita and Kiuchi, 2024); *cry1Δ^8^* alters circadian adult eclosion rhythm (Tobita and Kiuchi, 2024).	No direct hatching/eclosion assays are reported in the available studies.
Developmental/endocrine regulation	*Cry1^−/−^* delays larval/pupal development and affects PTTH-20E and JH-related pathways (Qiu et al., 2023).	No data are reported in the available studies.
Diapause regulation	*cry1^Δ8^* alters photoperiodic diapause responses (Tobita and Kiuchi, 2024); *Δcry1_5317* does not significantly affect temperature-dependent diapause induction under DD conditions (Homma et al., 2022).	*Δcry2_5731* disrupts temperature-dependent diapause induction (Homma et al., 2022).

Transcriptomic analyses during hatching showed phase-specific changes in clock gene expression in *Bmcry1* mutants, together with altered hatching-associated and neuroendocrine outputs. These included reduced expression and activity of chitinase and hatching enzyme-like protein, involved in chorion degradation, as well as impaired expression of short neuropeptide F, prothoracicotropic hormone and corazonin, a neuropeptide also involved in the diapause hormone pathway (Yuan et al., 2023). These data suggest that BmCRY1 contributes to the coordination of light-dependent hatching-associated and neuroendocrine outputs (Yuan et al., 2023).

A role for BmCRY1 in adult eclosion was shown using an independent CRISPR/Cas9-mediated *Bmcry1* knockout line (Tobita and Kiuchi, 2024). In DD, wild-type moths retained rhythmic eclosion, while mutants were arrhythmic.

The loss of rhythmicity in DD in *Bmcry1* knockouts contrasts with *Drosophila cry* mutants, which retained circadian locomotor rhythms in DD (Stanewsky et al., 1998). Yuan et al. (2023) implicated BmCRY1 in photoperiodic entrainment and clock oscillation during hatching. While not excluding a circadian clock impairment, Tobita and Kiuchi (2024) suggested that BmCRY1 loss could affect photic entrainment, thereby desynchronizing individual pupal rhythms.

The relevance of BmCRY1 light-input function became clear in diapause assays. Initially, Homma and colleagues showed that *Bmcry1* knockout females followed the wild-type temperature-dependent response under DD conditions, producing diapause eggs under warm conditions and non-diapause eggs under cold conditions (Homma et al., 2022). Subsequently, it was demonstrated that BmCRY1 is required for photoperiodic modulation of diapause (Tobita and Kiuchi, 2024). In LD, *Bmcry1* knockout females lost the photoperiodic response during the larval sensitive period, with 80% producing diapause eggs even under long-day conditions, while wild-type females produced non-diapause eggs. During the embryonic sensitive period at 18 °C, knockout females produced only non-diapause eggs irrespective of LL or DD exposure. In both cases the knockout reproduced the diapause phenotype of wild-type females reared in DD, indicating that BmCRY1 conveys photoperiodic information to the diapause pathway rather than executing the diapause decision itself (Tobita and Kiuchi, 2024).

In contrast to BmCRY1, functional evidence for BmCRY2 remains limited to temperature-dependent diapause induction. In DD, *Bmcry2* knockout females showed reduced diapause-egg production under warm conditions, broadly resembling the phenotype of core clock gene mutants, although with a less pronounced effect (Homma et al., 2022). This contrasts with *Bmcry1* mutants, which retained the wild-type temperature-dependent response, and suggests that BmCRY2 contributes to the clock-dependent branch required for temperature-dependent diapause induction.

However, the molecular role of BmCRY2 in the *B. mori* clockwork remains unresolved. Phylogeny and domain conservation support its classification as a CRY2-type clock component (**Figure 1**; **Table 1**), but *Bmcry2* mutants have not yet been analyzed for embryonic hatching or adult eclosion rhythms, and its predicted transcriptional repressor role has not been directly tested in *B. mori*. Thus, BmCRY2 is best interpreted as a likely clock-network component involved in diapause regulation, while its contribution to circadian outputs and position within the *B. mori* TTFL remain open questions.

## 3 Discussion

### 3.1 From CRY-dependent clock inputs to endocrine diapause output

The available mutant evidence suggests that BmCRY1 and BmCRY2 act at different entry points along the clock-diapause axis, but both routes are likely to converge, directly or indirectly, on the neuroendocrine pathway that determines embryonic diapause. In *B. mori*, diapause hormone (DH) secretion is controlled by a brain-suboesophageal ganglion pathway in which GABAergic signaling modulates corazonin-producing neurons, which in turn regulate DH-producing neurosecretory cells (Cui et al., 2021; Tsuchiya et al., 2021; Shimizu, 2024). This pathway provides the likely point of convergence for distinct CRY-dependent inputs, with BmCRY1 contributing primarily to photoperiodic light input and BmCRY2 potentially contributing to the clock-related branch of temperature-dependent diapause induction.

Temperature-dependent diapause involves BmTRPA1, a thermosensitive TRP channel proposed to act as a molecular switch (Sato et al., 2014; Yokoyama et al., 2021; Homma et al., 2022). At warm diapause-inducing temperatures (e.g., 25 °C), increased expression of the GABA transporter *GAT* is thought to remove GABA from the synaptic cleft and relieve inhibition of corazonin-producing neurons. Corazonin then promotes DH release, leading to diapause-egg production. Conversely, at cooler temperatures (e.g., ≤ 20 °C), persistent GABAergic inhibition suppresses this endocrine output (Tsuchiya et al., 2021; Shimizu, 2024). Clock genes intersect with this pathway upstream of GABAergic and DH signaling. Disruption of *Bmper*, *Bmtim*, *BmClk*, *Bmcyc* or *Bmcry2* reduces temperature-dependent diapause induction, and injection of picrotoxin, an antagonist of ionotropic GABA receptors, or synthetic DH restores diapause production in these mutant backgrounds, indicating that the downstream GABAergic and DH pathways remain functional (Homma et al., 2022). Moreover, *Bmper* disruption upregulates the GABA receptor *GRD* and downregulates the GABA transporter *GAT*, raising GABA content in the brain-suboesophageal ganglion complex and delaying DH release. Recently, *Bmcyc* knockout was shown to reduce corazonin expression and impair DH release (Zheng et al., 2025). These findings support a link between core clock components and the GABAergic-corazonin-DH pathway controlling DH release (Cui et al., 2021; Shimizu, 2024; Zheng et al., 2025).

Within this scheme, BmCRY1 appears to act upstream in the photoperiodic branch of diapause regulation. The *Bmcry1/Bmtim* double-mutant phenotype follows the *Bmtim* mutant phenotype, with females producing non-diapause eggs irrespective of photoperiod, placing BmTIM downstream of BmCRY1 in the photoperiodic diapause pathway (Tobita and Kiuchi, 2024). This supports a model in which BmCRY1 conveys photic information to the clock through a pathway that requires BmTIM (Tobita and Kiuchi, 2024). The model is consistent with the established role of *D. melanogaster* CRY in light-dependent TIM destabilization (Ceriani et al., 1999), although direct biochemical evidence for light-induced BmTIM degradation in *B. mori* is still lacking.

By contrast, the position of BmCRY2 in the clock-diapause axis remains less well defined. Although *Bmcry2* mutants show defects in temperature-dependent diapause induction, this phenotype cannot yet be linked to a defined downstream step in the GABAergic-DH pathway (Homma et al., 2022). Indeed, in 4^th^ day pupae, hemolymph DH levels were not significantly affected in *Bmcry2* mutants, despite their reduced diapause-egg production under warm DD conditions, suggesting that the precise step connecting BmCRY2 to the endocrine diapause pathway remains unresolved. Moreover, direct repression of BmCLK/BmCYC-mediated transcription by BmCRY2 has not yet been demonstrated, and *Bmcry2* mutants have not been evaluated for hatching or eclosion rhythms. Together, these observations suggest that neither BmCRY1 nor BmCRY2 act directly on DH release. Instead, the two CRYs may influence diapause through distinct clock-related inputs that ultimately impinge on the neuroendocrine cascade leading to maternal diapause commitment.

### 3.2 Emerging perspectives and future directions

This model raises several questions about how CRY-dependent inputs are integrated with other photoreceptive, clock, and endocrine mechanisms. For BmCRY1, an important open issue is its relationship with opsin-based photoreception. Boceropsin remains a candidate cerebral photoperiodic receptor, and the incomplete loss of photoperiodic responsiveness in *Bmcry1* mutants suggests that other brain photoreceptors may also contribute to diapause-relevant light input (Shimizu et al., 2001; Shimizu, 2024; Tobita and Kiuchi, 2024). Combining *Bmcry1* loss of function with disruption of candidate cerebral opsins, together with rescue or cell-specific manipulations, could demonstrate whether BmCRY1 acts as the main photic input or cooperates with opsin-dependent pathways. For BmCRY2, future work should define its role in clock-controlled phenotypes and its position within the *B. mori* clock network, including its potential repression of BmCLK/BmCYC-mediated transcription, binding partners, transcriptional targets, and tissue-specific expression. Established *B. mori* transgenic tools, including GAL4/UAS-based tissue-targeted expression, could support tissue-specific rescue, targeted knock-down and reporter analyses in clock and neuroendocrine circuits (Imamura et al., 2003; Kobayashi et al., 2011; Hara et al., 2017).

A further challenge is to understand how transient environmental information perceived through CRY/clock-related pathways is converted into a stable maternal and embryonic diapause program. In *B. mori*, this transition ultimately involves DH signaling to the ovary, activation of diapause hormone receptor (DHR)-dependent metabolic changes, including trehalose-to-glucose conversion and glycogen accumulation, and embryonic arrest at the late gastrula stage (Yamashita and Hasegawa, 1985; Su et al., 1994; Homma et al., 2006; Kamei et al., 2011; Cui et al., 2021). However, the molecular mechanisms stabilizing this developmental state downstream of CRY/clock and neuroendocrine inputs remain unclear. Epigenetic and post-transcriptional mechanisms provide plausible candidates, as they could help maintain long-lasting developmental information after the initial photoperiodic or thermal cue has been perceived. These mechanisms have been implicated in insect diapause and photoperiodic responses, and miRNA-mediated regulation of clock genes has been proposed during embryonic diapause induction in *B. mori* (Reynolds, 2017; Liu et al., 2021; Shimizu, 2024). More generally, circadian transcription is closely connected with chromatin dynamics, and recent comparative work indicates that clock proteins can cooperate with chromatin modifiers and RNA-regulatory pathways to shape rhythmic transcriptional and post-transcriptional states (Liu et al., 2026). Particularly relevant from a diapause perspective, a recent study in *Harmonia axyridis* showed that CLK and CYC regulate reproductive diapause through interaction with the NuA4/TIP60 histone acetyltransferase complex and juvenile hormone biosynthesis, illustrating a link between clock proteins, chromatin-level regulation and endocrine seasonal responses (Gao et al., 2025). Although no direct evidence currently links BmCRY1 or BmCRY2 to chromatin- or RNA-based regulation in *B. mori*, future work should test whether CRY-dependent pathways intersect with molecular memory mechanisms stabilizing maternal diapause commitment.

Strain variation is relevant both to understanding diapause plasticity and to sericulture. Domestic *B. mori* strains differ in their photoperiodic and thermal diapause responses as a result of natural variation and long-term selection during domestication (Yamashita and Hasegawa, 1985; Shimizu, 2024). Studies in *Drosophila*, where diapause occurs at the adult reproductive stage, support a contribution of natural variation in clock genes to photoperiodic diapause (Sandrelli et al., 2007b; Tauber et al., 2007; Yamada and Yamamoto, 2011). Recently, in *B. mori* a naturally occurring one-base-pair deletion on chromosome Z selectively disrupting a BmCYC isoform was shown to be a major determinant of the non-diapause phenotype in polyvoltine strains (Zheng et al., 2025). Because CRY-diapause phenotypes have so far been characterized in bivoltine strains, comparisons across voltinism backgrounds will be needed to determine how the genetic architecture of voltinism modulates CRY- and clock-dependent environmental responses.

Polymorphisms in *Bmcry1*, *Bmcry2*, or other clock-related genes may also contribute to differences among *B. mori* strains. If robustly associated with diapause responses, such variants could serve as early markers of egg diapause trajectory. More generally, defining how BmCRY1 and BmCRY2, within the circadian clock network, modulate maternal responses to photoperiod and temperature could help optimize rearing conditions to produce diapause eggs for prolonged storage or non-diapause eggs for continuous rearing.

Finally, non-diapause eggs offer a biotechnological opportunity, particularly for transgenesis and genome editing, which require hundreds to thousands of fresh eggs. Although DH-based pupal injections can alter diapause, they are impractical for routine strain management (Grenier et al., 2004). Targeting CRY-dependent diapause pathways may represent an alternative or complementary strategy for obtaining non-diapause eggs. However, phenotype stability and possible effects on strain performance will need to be evaluated before practical application in silkworm biotechnology.
